# Association Between Serum Apolipoprotein B and Bone Mineral Density and the Effects of Cardiovascular Disease Mediation: Results From the NHANES 2011–2016 and a Mendelian Randomization Study

**DOI:** 10.31083/RCM31395

**Published:** 2025-05-23

**Authors:** Aochuan Sun, Yiduo Chen, Yang Wu, Zhuangzhuang Li, Jingchun Zhang, Zhengtang Liu

**Affiliations:** ^1^Graduate School, Beijing University of Chinese Medicine, 100029 Beijing, China; ^2^The Department of Geriatrics, Xiyuan Hospital, China Academy of Chinese Medical Sciences, 100091 Beijing, China; ^3^National Clinical Research Center for Chinese Medicine Cardiology, Xiyuan Hospital, China Academy of Chinese Medical Sciences, 100091 Beijing, China

**Keywords:** apolipoprotein B, bone mineral density, National Health and Nutrition Examination Survey, Mendelian randomization, cardiovascular disease, mediating factor

## Abstract

**Background:**

Previous studies have indicated that blood lipids can influence skeletal health. However, limited research exists on the impact of serum apolipoprotein B (ApoB) on bone mineral density (BMD); meanwhile, it remains unclear to what extent cardiovascular disease plays in mediating this process.

**Methods:**

Therefore, we conducted a cross-sectional analysis involving 2930 participants from the National Health and Nutrition Examination Survey (NHANES) database to explore the relationship between serum ApoB and total body BMD (TB-BMD) and lumbar spine BMD (LS-BMD). We employed a two-step, two-sample Mendelian randomization (MR) analysis using genetic instruments to investigate causality and assess the mediating effects of six cardiovascular diseases.

**Results:**

Multivariable linear regression models demonstrated an inverse linear association between serum ApoB and TB-BMD (β = –0.26, 95% confidence interval (CI): –0.41 to –0.12, *p* < 0.001; *p* for non-linearity = 0.771) and LS-BMD (β = –0.53, 95% CI: –0.75 to –0.31, *p* < 0.001; *p* for non-linearity = 0.164). The primary analysis utilized the multiplicative random effects inverse variance weighted (IVW-MRE) method for the two-sample MR analysis. The results demonstrated a causal relationship between serum ApoB with TB-BMD (β = –0.0424, 95% CI: –0.0746 to –0.0103; *p* = 0.0096) and LS-BMD (β = –0.0806, 95% CI: –0.1384 to –0.0229; *p* = 0.0062). The two-step MR analysis indicated heart failure as a mediating factor in the causal relationship between serum ApoB and TB-BMD, with a mediation proportion of 18.69%.

**Conclusions:**

The results of this study support that lowering serum ApoB levels could enhance BMD while preventing the occurrence of heart failure might reduce the harm caused by the decrease in BMD due to elevated ApoB levels.

## 1. Introduction

Osteoporosis is a widespread condition that impacts a significant portion of the global population [1]. It is characterized by a reduction in bone mass and the degradation of bone tissue microstructure, which significantly increases the risk of a fracture [2]. The diagnosis is established by assessing bone mineral density (BMD) through dual-energy X-ray absorptiometry (DXA) scans [3]. By the year 2030, it is anticipated that the prevalence of osteoporosis or low bone mass will increase from approximately 53 million to over 70 million [4]. Therefore, it is imperative to explore novel biomarkers for bone health.

The relationship between serum lipids and BMD remains inconclusive, with inconsistent findings reported in previous studies [5, 6]. A study suggested that serum ApoB in serum lipids may have a negative correlation with BMD, indicating its potential as an indicator of bone health [7]. In addition, previous studies have mostly ignored the effect of serum apolipoprotein B (ApoB) on bone health. Serum ApoB is considered to be a key indicator of cardiovascular diseases such as atherosclerosis [8, 9, 10]. A previous study indicated a nonlinear relationship between femoral bone mineral density and the risk of cardiovascular disease [11]. Another study reported prospective associations between BMD and cardiovascular diseases, including atrial fibrillation, acute myocardial infarction, stroke, and heart failure [12]. Previous studies have suggested that there is an associations between osteoporosis and deaths from cardiovascular disease [13, 14, 15]. A major limitation in studying the relationship between ApoB and BMD is that hyperlipidemia often coexists with conditions that may affect bone health, such as cardiovascular diseases. Consequently, when an association is observed, it is challenging to distinguish the effects of hyperlipidemia from those of cardiovascular diseases. From 1990 to the subsequent two decades, the prevalence of cardiovascular diseases doubled, with the number of cases increasing by 252 million and deaths rising by 6.5 million [16, 17]. Therefore, serum ApoB may represent a useful indicator for changes in BMD. In addition, investigating the role of cardiovascular disease could further elucidate the pathogenesis of osteoporosis and reveal relevant targets for reducing the risk of osteoporosis.

Mendelian randomization (MR) analysis is a method that employs genetic data to clarify causal associations between exposures and outcomes. Since genetic alleles are randomly assigned during meiosis and not influenced by environmental factors, the genetic associations identified in MR analyses are less susceptible to confounding bias and the potential for reverse causation [18]. Consequently, MR studies offer a valuable complementary approach for investigating the causal link between serum ApoB and BMD.

Currently, there is limited research exploring the correlation between ApoB and BMD, as well as the potential a causal relationship and underlying mediating pathways. To comprehensively investigate the association between ApoB and BMD, we initially conducted a cross-sectional study using the National Health and Nutrition Examination Survey (NHANES) database. Subsequently, we performed MR analysis to assess its causal relationship at the genetic level. We were particularly interested in evaluating the extent to which various cardiovascular diseases mediated these relationships.

## 2. Materials and Methods

### 2.1 Study Framework

**Fig. 1.**
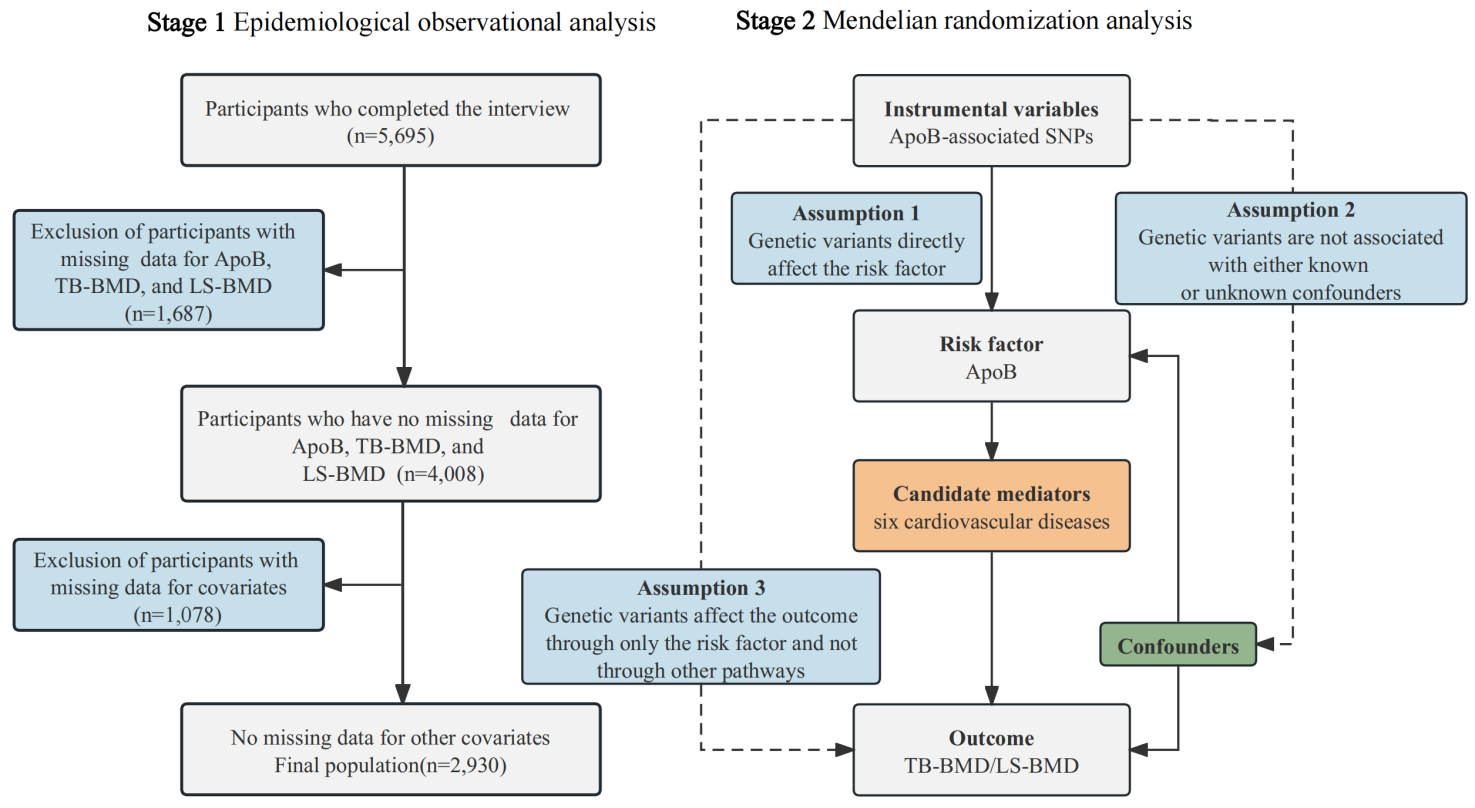
**Study framework**. BMD, bone mineral density; SNPs, single-nucleotide polymorphisms; ApoB, apolipoprotein B; TB-BMD, total body BMD; LS-BMD, lumbar spine BMD.

This study consisted of two phases: epidemiological observational analysis and Mendelian randomization analysis, as illustrated in Fig. 1. In the initial phase, we utilized data from the NHANES database to perform a multivariable linear regression analysis. This analysis sought to investigate the correlation between serum ApoB with total body BMD (TB-BMD) and lumbar spine BMD (LS-BMD). During the second phase, MR analysis was conducted using data from summary statistics obtained from genome-wide association studies (GWAS). This study aimed to assess the causal relationship between ApoB and BMD at various sites, including TB-BMD, LS-BMD, and femoral neck BMD (FN-BMD). Subsequently, a further evaluation was conducted to assess the mediating role of six cardiovascular diseases (stroke, coronary artery disease, atrial fibrillation, heart failure, venous thromboembolism, and peripheral atherosclerosis) in the causal relationship between ApoB and BMD. All statistical tests were conducted using a two-tailed approach at a significance level set to *p* < 0.05. Following relevant policies, the Institutional Review Board determined that a review was not required for the secondary analysis. All analyses were performed using R Statistical Software (Version 4.2.2, http://www.R-project.org, The R Foundation) and Free Statistics analysis platform (Version 1.9, Beijing, China, http://www.clinicalscientists.cn/freestatistics). We utilized the functions provided by these packages and their related functions to perform our analyses. Our study followed the guidelines outlined in the STROBE and STROBE-MR.

### 2.2 Epidemiological Observational Analysis

#### 2.2.1 Study Population

The NHANES study employed a comprehensive multistage survey method that incorporated stratification to enhance precision and accuracy in its outcomes. The research received ethical clearance from the National Center for Health Statistics Research Ethics Review Board, and prior to participation, all individuals provided written informed consent [19]. We acquired data from adult participants (20–59 years) in NHANES from 2011–2016. We excluded participants from the study if they had incomplete data on serum ApoB, TB-BMD, LS-BMD, or covariates (Fig. 1).

#### 2.2.2 Outcome Ascertainment and Exposure Measurement

The BMD measurements were performed utilizing fan-beam densitometers (Hologic QDR-4500A, Bedford, MA, USA) through DXA scans [20]. ApoB within human serum can engage in an immunochemical reaction, resulting in the creation of immune complexes. These complexes induce alterations in the intensity of scattered light as it traverses through them, and this modified intensity is quantified using a Siemens Prospec chemistry analyzer, enabling precise determination of the ApoB concentration [7]. For further detailed information, please consult the official NHANES website.

#### 2.2.3 Assessment of Other Covariates

We selected these confounders on the basis of judgment, previous scientific literature, all significant covariates in the univariate analysis, or their associations with the outcomes of interest or a change in effect estimate of more than 10% [7, 21]. Standardized questionnaires devised by NHANES researchers collected demographic data, including gender, age, race, education level, poverty-to-income ratio (PIR), marital status, drinking status (determined by whether participants consumed at least 12 alcoholic drinks annually), and smoking status (assessed based on whether participants had smoked a minimum of 100 cigarettes in their lifetime), and physical activity (min/week). Body mass index (BMI) was calculated from weight and height (kg/m^2^). Co-morbidities diagnosed by physicians, such as liver disease and cancer or malignancies, were determined based on self-reporting by participants. The levels of serum urea nitrogen, serum phosphorus, serum total protein, and serum uric acid were determined using standardized protocols. The intake of magnesium, calcium, sodium, and potassium was obtained through 24-hour dietary recalls conducted by trained interviewers at the mobile examination center.

#### 2.2.4 Statistical Analysis

Categorical variables were presented using frequencies, whereas continuous variables that followed a normal or skewed distribution were expressed as the mean or median, respectively. We employed One-Way ANOVA for normally distributed data, the Kruskal-Wallis H test for skewed distributions, and the chi-square test for categorical variables to compare the clinical characteristics across ApoB quartiles. We assessed the relationship between serum ApoB levels and TB-BMD as well as LS-BMD using linear regression models, presenting regression coefficients (β) along with their respective 95% confidence intervals [CI]. We utilized both unadjusted and multivariable-adjusted models. In our study, Model 1 was adjusted for gender, age, race, education level, marital status, PIR, and BMI. Model 2 was adjusted for Model 1 + drinking status, smoking status, physical activity, liver condition, cancer or malignancy. Model 3 was completely adjusted, including Model 1 + Model 2 + serum urea nitrogen, serum phosphorus, serum total protein, serum uric acid, calcium, magnesium, sodium, and potassium. To demonstrate the stability of the results and assess potential non-linearity, ApoB was divided into quartiles, and trend *p*-values were analyzed. In Model 3, we further evaluated the dose-response relationship using a restricted cubic spline (with four knots positioned at the 5th, 35th, 65th, and 95th percentiles of ApoB levels). A multicollinearity check was performed to evaluate the collinearity between the covariates and ApoB. In addition, we conducted multiple sensitivity analyses to assess the robustness of our findings. Subgroup analyses were performed to evaluate potential variations in the relationship between ApoB and BMD, stratified by age (using 45 years as the cutoff), gender, drinking status, and smoking status. Interactions between subgroups were tested using likelihood ratio tests. To further evaluate the robustness of the results, participants with extreme ApoB levels (outside the range of mean ± 3 standard deviations) were excluded. For handling missing data in the study, we employed a multiple imputation approach based on 5 repetitions and the chained equations method using the “mice” package in R, aiming to maximize statistical power and minimize potential bias arising from missing data.

### 2.3 MR Study

#### 2.3.1 Study Design

The present MR study was composed of two steps (Fig. 1). In the first step, a two-sample univariable mendelian randomization (UVMR) approach was employed to assess the causal relationships between serum ApoB levels and BMD (TB-BMD, LS-BMD, and FN-BMD). The results from the UVMR analysis suggested a causal relationship between ApoB and TB-BMD as well as LS-BMD, while no causal relationship was observed between ApoB and FN-BMD. In the second step, both UVMR and multivariable mendelian randomization (MVMR) were employed to screen for mediators of the association between ApoB and the correlation of TB-BMD as well as LS-BMD among six cardiovascular diseases (stroke, coronary artery disease, atrial fibrillation, heart failure, venous thromboembolism, and peripheral atherosclerosis). Subsequently, a two-step MR analysis was performed to compute the mediating effects.

#### 2.3.2 Data Sources

We utilized summary statistics obtained from extensive genome-wide association studies (GWAS) or meta-analyses of GWAS data to perform estimation of genetic correlations and MR analysis. The summary statistics for the serum ApoB dataset were derived from the UK Biobank GWAS summary statistics, which included 435,744 participants of European ancestry. Basic quality control measures were applied to the serum ApoB trait, including the exclusion of extreme outliers, stratification by gender and menopausal status, inverse normal transformation, removal of covariates, and subsequent reapplication of inverse normal transformation [22].

The summary statistics for TB-BMD were obtained from a meta-analysis of GWAS involving 56,284 participants. TB-BMD was assessed using DXA scans following standard manufacturer protocols [23]. The data is publicly available from the Genetic Factors for Osteoporosis (GeFOS) consortium [24]. The most extensive GWAS dataset for BMD assessed using DXA was acquired from GEFOS, encompassing FN-BMD data from 32,735 European participants and LS-BMD data from 28,498 European participants [25]. Additionally, we selected six cardiovascular diseases including stroke, coronary artery disease, atrial fibrillation, heart failure, venous thromboembolism, and peripheral atherosclerosis as candidate mediators. The GWAS datasets for these conditions were obtained from various organizations or consortia, namely MEGASTROKE, the UK Biobank study, Center for Statistical Genetics, Heart Failure Molecular Epidemiology for Therapeutic Targets, and the FinnGen consortium. All participants included in these studies shared European ancestry. Detailed information can be found in (**Supplementary Table 1**).

#### 2.3.3 Instrumental Variables Selection

The MR analysis is based on three fundamental assumptions [26]. The initial assumption necessitates that instrumental variables (IVs) exhibit an association with the risk factor. Second, these genetic variants should not be connected to confounding factors. Third, IVs should influence the outcome risk solely through the risk factor, without involving other pathways. To meet these three assumptions in the statistical analysis, we conducted quality control procedures to select appropriate IVs. (1) We opted for single-nucleotide polymorphisms (SNPs) meeting the genome-wide significance threshold (*p* ≤ 5 × 10^-8^), ensuring their strong association with the exposure of interest. (2) We excluded palindromic SNPs due to their uncertain direction effect. Subsequently, we performed a linkage disequilibrium (LD) clumping procedure to preserve independent SNPs, setting an r^2^ threshold of 0.001 and a clumping window size of 10,000 kb. Any SNPs exhibiting an r^2^ value exceeding 0.01 with the lead SNP within 1000 kb were eliminated from consideration. (3) We assessed the robustness of IVs using the F statistic (detailed formulae are shown in **Supplementary Table 2**). SNPs having an F-statistic < 10 are classified as weak IVs and were omitted from the analysis due to their potential to introduce bias into the results. (4) We corrected for the influence of ambiguous SNPs with non-concordant alleles and palindromic SNPs with ambiguous strands when possible. When correction was not feasible, we excluded these ambiguous and palindromic SNPs from the initially selected instrumental SNPs during the harmonization process [27]. Following a stringent screening process, the selected SNPs were employed as IVs in subsequent MR analyses.

#### 2.3.4 Statistical Analysis

In the two-sample UVMR analysis, we separately evaluated the causal relationships of ApoB with TB-BMD and LS-BMD, denoting the estimated coefficients as β0. We then conducted a two-step MR analysis to explore whether the six cardiovascular diseases could mediate the causal relationship between ApoB with TB-BMD and LS-BMD. The first step involved employing UVMR to assess the causal effects of ApoB on each cardiovascular disease, denoted as β1. To ensure the validity of the mediating effects, UVMR was employed to evaluate the absence of reverse causation between ApoB and each cardiovascular disease. The second step entailed the concurrent use of UVMR and MVMR to assess the causal relationship of each cardiovascular disease with BMD, both before and after adjustment for ApoB. The estimated values from MVMR were noted as β2. The proportion of mediation was calculated using the formula β1 × β2/β0, and the 95% CI for this mediation effect was computed using the delta method [28].

For UVMR, we assessed the potential causal associations between the exposure variable and the outcome variable using the fixed effects inverse variance weighted (IVW-FE) method. Additionally, we employed three other methods for additional analysis, including Weighted Median, MR Egger, and MR pleiotropy residual sum and outlier (MR-PRESSO). Sensitivity analyses were conducted to validate and ensure the reliability and stability of the results. These analyses encompassed various tests, including the assessment of heterogeneity using Cochrane’s Q test, as well as pleiotropy testing through the MR Egger intercept test [29]. In cases where heterogeneity was detected (*p* < 0.05), the multiplicative random effects IVW (IVW-MRE) method was employed to evaluate the causal effect [30]. The IVW method served as the primary analytical approach in this study. Additionally, we employed the Weighted Median method, renowned for its resilience to potentially flawed instrumental variables [31]. Moreover, we utilized the MR Egger method, proficient at detecting and mitigating bias stemming from directional pleiotropy, operating under the assumption of the instrument strength independent of direct effect (InSIDE) principle [29]. To identify and address the influence of outlier SNPs exhibiting pleiotropic effects, we utilized the MR-PRESSO method, assessing their impact on causal relationships [32]. Finally, we utilized UVMR to assess the presence of reverse causation between ApoB with TB-BMD and LS-BMD. The MVMR analysis was conducted using the IVW method. The odds ratio (OR), β coefficient, or proportions are presented along with their respective 95% CI to indicate the magnitude of the effects. We applied the Bonferroni correction to address multiple comparisons, determining the critical *p* value according to the number of exposures and outcomes [33].

## 3. Results

### 3.1 Epidemiological Observational Analysis

#### 3.1.1 Baseline Characteristics

This study involved 5695 prospective participants (20–59 years) from NHANES (2011–2016) who completed interviews. We excluded participants with missing data for ApoB, TB-BMD, and LS-BMD (n = 1687). Finally, 2930 participants with complete datasets were included (Fig. 1). The mean participant age (years) was 38.9 ± 11.5, and 1530 (52.2%) were men. The mean serum ApoB, TB-BMD, and LS-BMD were 91.0 ± 25.7 mg/dL, 1111.7 ± 108.0 mg/cm^2^, and 1031.0 ± 149.6 mg/cm^2^, respectively. The comprehensive population characteristics categorized by serum ApoB quartiles can be found in **Supplementary Table 3**.

#### 3.1.2 Association Between ApoB With TB-BMD and LS-BMD

**Fig. 2.**
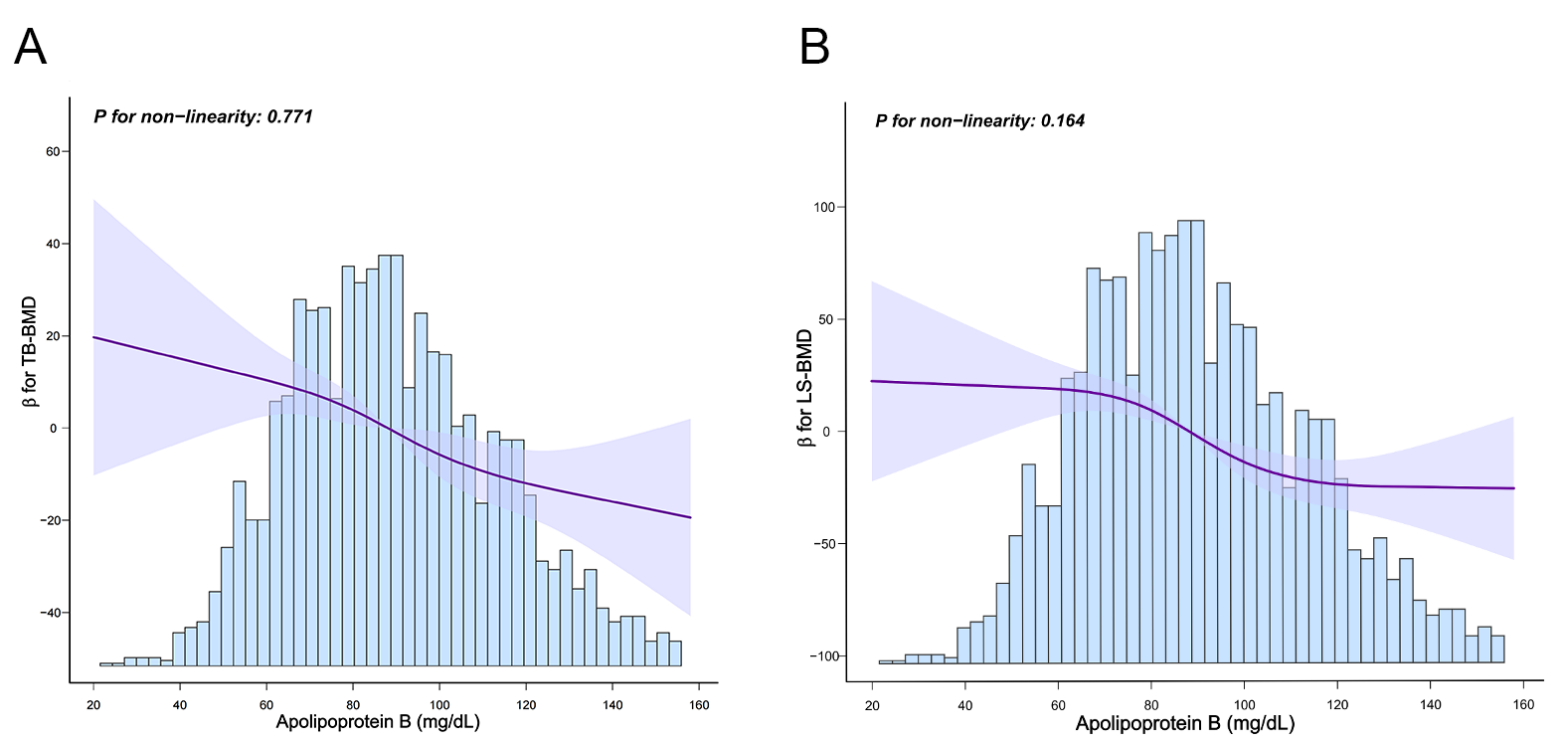
**Restricted cubic spline model of ApoB with TB-BMD and LS-BMD**. (A) Restricted cubic spline model of ApoB and TB-BMD. (B) Restricted cubic spline model of ApoB and LS-BMD. Solid line represents β; lightly shaded area represents 95% CI.

The results of the univariate analysis for TB-BMD and LS-BMD are presented in **Supplementary Table 4**. Following multivariable adjustment, a significant association was observed between ApoB and TB-BMD. Model 3 revealed an adjusted β of –0.26 (95% CI: –0.41 to –0.12, *p* < 0.001) for TB-BMD when ApoB was considered a continuous variable. When compared to participants within the lowest ApoB quartile (20–72 mg/dL), the adjusted β for the serum ApoB and TB-BMD in the 4th quartile (107–260 mg/dL) was –17.41 (95% CI: –28.12 to –6.70, *p* < 0.001) in Model 3. Similarly, ApoB was also significantly correlated with LS-BMD. In Model 3, when ApoB was handled as a continuous variable, the adjusted β associated with LS-BMD was –0.53 (95% CI: –0.75 to –0.31, *p* < 0.001). Compared to participants within the lowest ApoB quartile, those in the 4th quartile exhibited an adjusted β for serum ApoB and LS-BMD of –37.12 (95% CI: –53.21 to –21.03, *p* < 0.001) in Model 3 (Table 1). There was a linear relationship between serum ApoB with TB-BMD (*p* for non-linearity = 0.771) and LS-BMD (*p* for non-linearity = 0.164) (Fig. 2). All VIFs were <2.0, indicating no significant multicollinearity. The subgroup analysis revealed no significant interactions in any of the stratified groups (**Supplementary Figs. 1,2**). After excluding extreme ApoB values, the results remained stable upon adjustment for all covariates (**Supplementary Table 5**). Missing data, as detailed in **Supplementary Table 6**, were addressed using multiple imputation, and the results remained robust following this procedure (**Supplementary Table 7**).

**Table 1.** **Association between ApoB with TB-BMD and LS-BMD in multivariable regression model**.

Variable		Unadjusted		Model 1		Model 2		Model 3	
		β (95% CI)	*p*-value	β (95% CI)	*p*-value	β (95% CI)	*p*-value	β (95% CI)	*p*-value
TB-BMD, (mg/cm^2^)									
	ApoB (mg/dL)	–0.21 (–0.37 to –0.06)	0.006	–0.28 (–0.42 to –0.13)	<0.001	–0.27 (–0.42 to –0.13)	<0.001	–0.26 (–0.41 to –0.12)	<0.001
	Q1 (20–72 mg/dL)	0 (Ref)		0 (Ref)		0 (Ref)		0 (Ref)	
	Q2 (73–88 mg/dL)	–1.72 (–12.8 to 9.36)	0.761	–1.57 (–11.46 to 8.32)	0.755	–1.33 (–11.21 to 8.54)	0.791	–0.81 (–10.70 to 9.07)	0.872
	Q3 (89–106 mg/dL)	–9.53 (–20.73 to 1.67)	0.096	–8.97 (–19.23 to 1.29)	0.087	–8.50 (–18.76 to 1.75)	0.104	–8.21 (–21.09 to 0.88)	0.119
	Q4 (107–260 mg/dL)	–13.55 (–24.65 to –2.44)	0.017	–18.15 (–28.67 to –7.63)	<0.001	–17.96 (–28.47 to –7.44)	0.001	–17.41 (–28.12 to –6.70)	0.001
	*p* for trend		<0.001		<0.001		<0.001		<0.001
LS-BMD, (mg/cm^2^)									
	ApoB (mg/dL)	–0.73 (–0.94 to –0.52)	<0.001	–0.55 (–0.76 to –0.33)	<0.001	–0.55 (–0.77 to –0.33)	<0.001	–0.53 (–0.75 to –0.31)	<0.001
	Q1 (20–72 mg/dL)	0 (Ref)		0 (Ref)		0 (Ref)		0 (Ref)	
	Q2 (73–88 mg/dL)	–17.07 (–32.32 to –1.83)	0.028	–11.01 (–25.83 to 3.81)	0.146	–10.86 (–25.67 to –3.96)	0.151	–10.01 (–24.87 to 4.85)	0.187
	Q3 (89–106 mg/dL)	–38.92 (–54.33 to –23.51)	<0.001	–28.19 (–43.56 to –12.82)	<0.001	–28.33 (–43.71 to –12.94)	<0.001	–27.78 (–43.26 to –12.31)	<0.001
	Q4 (107–260 mg/dL)	–52.44 (–67.72 to –37.17)	<0.001	–38.40 (–54.17 to –22.64)	<0.001	–38.77 (–54.55 to –22.99)	<0.001	–37.12 (–53.21 to –21.03)	<0.001
	*p* for trend		<0.001		<0.001		<0.001		<0.001

Ref, reference; PIR, poverty-to-income ratio; BMI, body mass index. Model 1 adjust for gender, age, race, education level, marital status, PIR, and BMI. Model 2 adjust for Model 1 + drinking status, smoking status, physical activity, liver condition, cancer or malignancy. Model 3 adjust for Model 1 + Model 2 + serum urea nitrogen, serum phosphorus, serum total protein, serum uric acid, calcium, magnesium, sodium, and potassium.

### 3.2 MR Study

#### 3.2.1 Causal Effects of Serum ApoB on TB-BMD, LS-BMD and FN-BMD

**Fig. 3.**
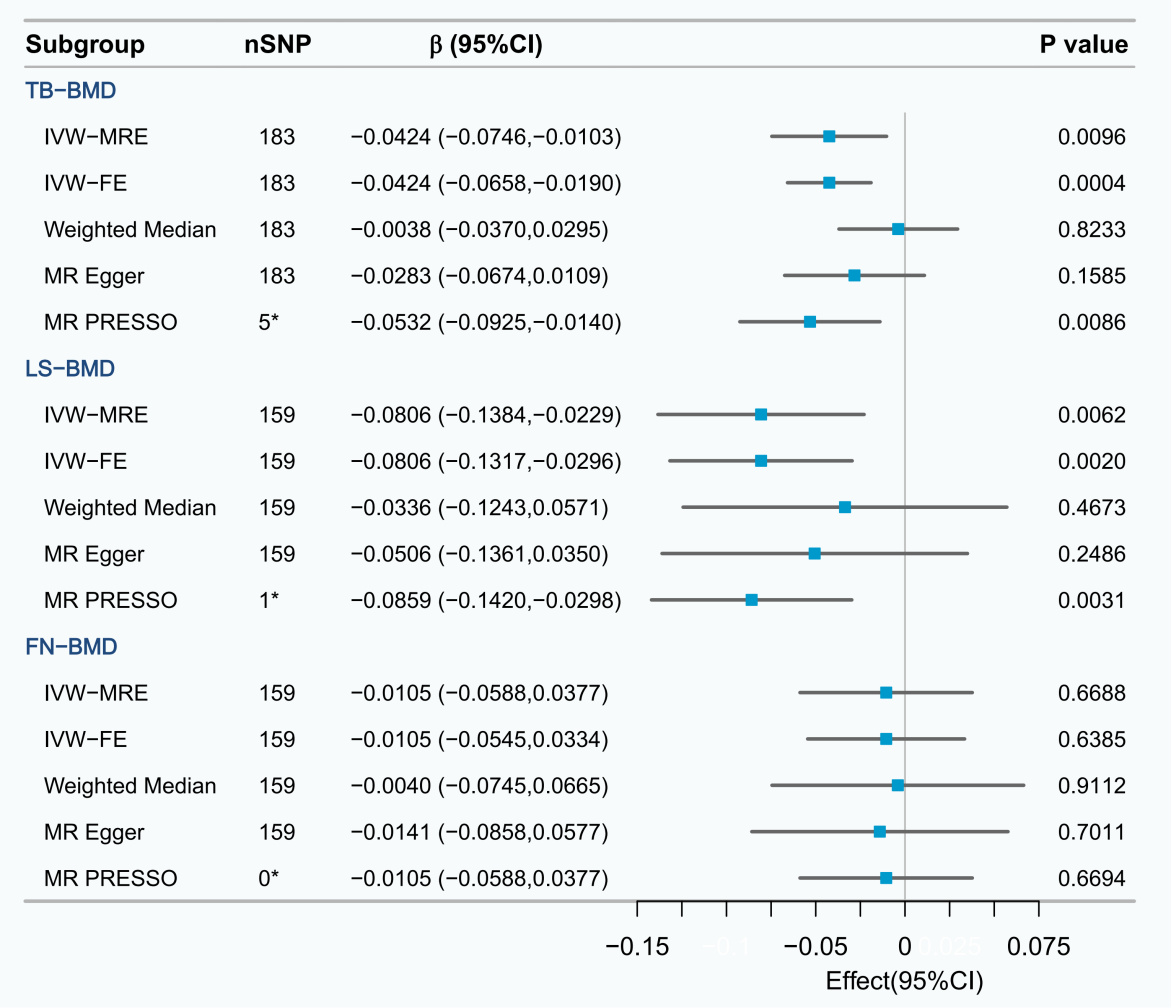
**UVMR analysis of the effect of serum ApoB on TB-BMD, LS-BMD and FN-BMD**. *The number of outliers; IVW-FE, fixed effects inverse variance weighted; IVW-MRE, multiplicative random effects inverse variance weighted; MR PRESSO, mendelian randomization pleiotropy residual sum and outlier; UVMR, univariable mendelian randomization; FN-BMD, femoral neck bone mineral density.

In the UVMR analysis, a causal relationship was observed between a 1-standard deviation (SD) increase in serum ApoB levels and a subsequent decrease in TB-BMD (β_I⁢V⁢W-F⁢E_ = –0.0424, 95% CI: –0.0658 to –0.0190, *p* = 0.0004) and LS-BMD (β_I⁢V⁢W-F⁢E_ = –0.0806, 95% CI: –0.1317 to –0.0296, *p* = 0.0020). The MR-PRESSO method confirmed the existence of the causal relationship, while the Weighted Median method and the MR Egger method demonstrated trends consistent with the study results. None of the methods indicated a causal relationship between ApoB and FN-BMD (Fig. 3). The detailed information of the SNPs is presented in **Supplementary Table 8**.

The calculations determined that the F-statistic of every SNP exceeded the threshold of 10 (**Supplementary Table 2**). Therefore, the likelihood of a weak IV affecting the results was relatively low. The sensitivity analysis revealed heterogeneity in the results (Cochrane’s Q test *p* < 0.05), but no evidence of pleiotropy (MR-Egger intercept test *p* > 0.05). The detailed results can be found in **Supplementary Table 9**. The subsequent application of the IVW-MRE method further confirmed the causal effect of ApoB on TB-BMD (β_I⁢V⁢W-M⁢R⁢E_ = –0.0424, 95% CI: –0.0746 to –0.0103, *p* = 0.0096) and LS-BMD (β_I⁢V⁢W-M⁢R⁢E_ = –0.0806, 95% CI: –0.1384 to –0.0229, *p* = 0.0062). In addition, we performed reverse association analyses of serum ApoB on TB-BMD, LS-BMD, and FN-BMD, and these analyses showed no evidence of reverse causality (**Supplementary Table 10**).

#### 3.2.2 Causal Effects of ApoB on Cardiovascular Diseases

In the UVMR, employing the IVW-FE method revealed a causal relationship between genetically determined serum ApoB levels (per 1-SD increase) and an elevated risk of all five cardiovascular diseases (except venous thromboembolism) (Table 2). All SNPs for each variable exhibit sufficient instrument strength, with F-statistics exceeding 10 (**Supplementary Table 2**). The MR-Egger intercept test indicated the absence of horizontal pleiotropy across all analyses (all *p* > 0.05). The Cochrane’s Q test indicated heterogeneity across all analyses (**Supplementary Table 11**). Therefore, employing the IVW-MRE method to assess causality revealed that serum ApoB levels were associated with stroke (OR_I⁢V⁢W-M⁢R⁢E_ = 1.1068, 95% CI: 1.0505 to 1.1662, *p* = 0.0001), coronary artery disease (OR_I⁢V⁢W-M⁢R⁢E_ = 1.3693, 95% CI: 1.2649 to 1.4822, *p* = 7.80 × 10^-15^), heart failure (OR_I⁢V⁢W-M⁢R⁢E_ = 1.0729, 95% CI: 1.0202 to 1.1284, *p* = 0.0062), and peripheral Atherosclerosis (OR_I⁢V⁢W-M⁢R⁢E_ = 1.2790 , 95% CI: 1.2620 to 1.4199, *p* = 4.88 × 10^-15^). In the reverse MR analysis, only a causal relationship between coronary artery disease and ApoB was observed (**Supplementary Table 10**).

**Table 2.** **Causal effects of serum ApoB on cardiovascular diseases**.

Cardiovascular diseases		nSNP	OR (95% CI)	*p* value
Stroke				
	IVW-MRE	179	1.1068 (1.0505, 1.1662)	0.0001
	IVW-FE	179	1.1068 (1.0695, 1.1455)	6.99 × 10^–⁢9^
	Weighted Median	179	1.0956 (1.0417, 1.1524)	0.0004
	MR Egger	179	1.1343 (1.0640, 1.2092)	0.0002
	MR PRESSO	5*	1.1142 (1.0647, 1.1661)	6.22 × 10^–⁢6^
Coronary artery disease				
	IVW-MRE	150	1.3693 (1.2649, 1.4822)	7.80 × 10^–⁢15^
	IVW-FE	150	1.3693 (1.3036, 1.4383)	5.27 × 10^–⁢36^
	Weighted Median	150	1.3621 (1.2307, 1.5076)	2.39 × 10^–⁢9^
	MR Egger	150	1.4684 (1.2866, 1.6759)	6.39 × 10^–⁢8^
	MR PRESSO	4*	1.3875 (1.2901, 1.4923)	3.30 × 10^–⁢15^
Atrial fibrillation				
	IVW-MRE	184	1.0297 (0.9878, 1.0734)	0.1668
	IVW-FE	184	1.0297 (1.0022, 1.0580)	0.0339
	Weighted Median	184	1.0148 (0.9768, 1.0542)	0.4499
	MR Egger	184	1.0541 (1.0023, 1.1085)	0.0418
	MR PRESSO	7*	1.0373 (1.0020, 1.0738)	0.0397
Heart failure				
	IVW-MRE	163	1.0729 (1.0202, 1.1284)	0.0062
	IVW-FE	163	1.0729 (1.0366, 1.1105)	6.05 × 10^–⁢5^
	Weighted Median	163	1.0114 (0.9644, 10607)	0.6394
	MR Egger	163	1.0694 (1.0062, 1.1366)	0.0323
	MR PRESSO	5*	1.1488 (1.0801, 1.2219)	1.91 × 10^–⁢5^
Venous thromboembolism				
	IVW-MRE	182	0.9301 (0.8555, 1.0112)	0.0894
	IVW-FE	182	0.9301 (0.8703, 0.9940)	0.0326
	Weighted Median	182	0.8917 (0.8072, 0.9851)	0.0241
	MR Egger	182	0.9048 (0.8160, 1.0032)	0.0591
	MR PRESSO	0*	0.9301 (0.8555, 1.0112)	0.0911
Peripheral atherosclerosis				
	IVW-MRE	181	1.2790 (1.2620, 1.4119)	4.88 × 10^–⁢5^
	IVW-FE	181	1.2620 (1.1595, 1.3735)	7.32 × 10^–⁢8^
	Weighted Median	181	1.0226 (0.8959, 1.1672)	0.7406
	MR Egger	181	1.2728 (1.1081, 1.4620)	0.0008
	MR PRESSO	4*	1.4196 (1.2432, 1.6210)	6.12 × 10^–⁢7^

*The number of outliers; OR, odds ratio.

#### 3.2.3 Mediation Effect of Heart Failure in the Association Between ApoB With TB-BMD 

**Fig. 4.**
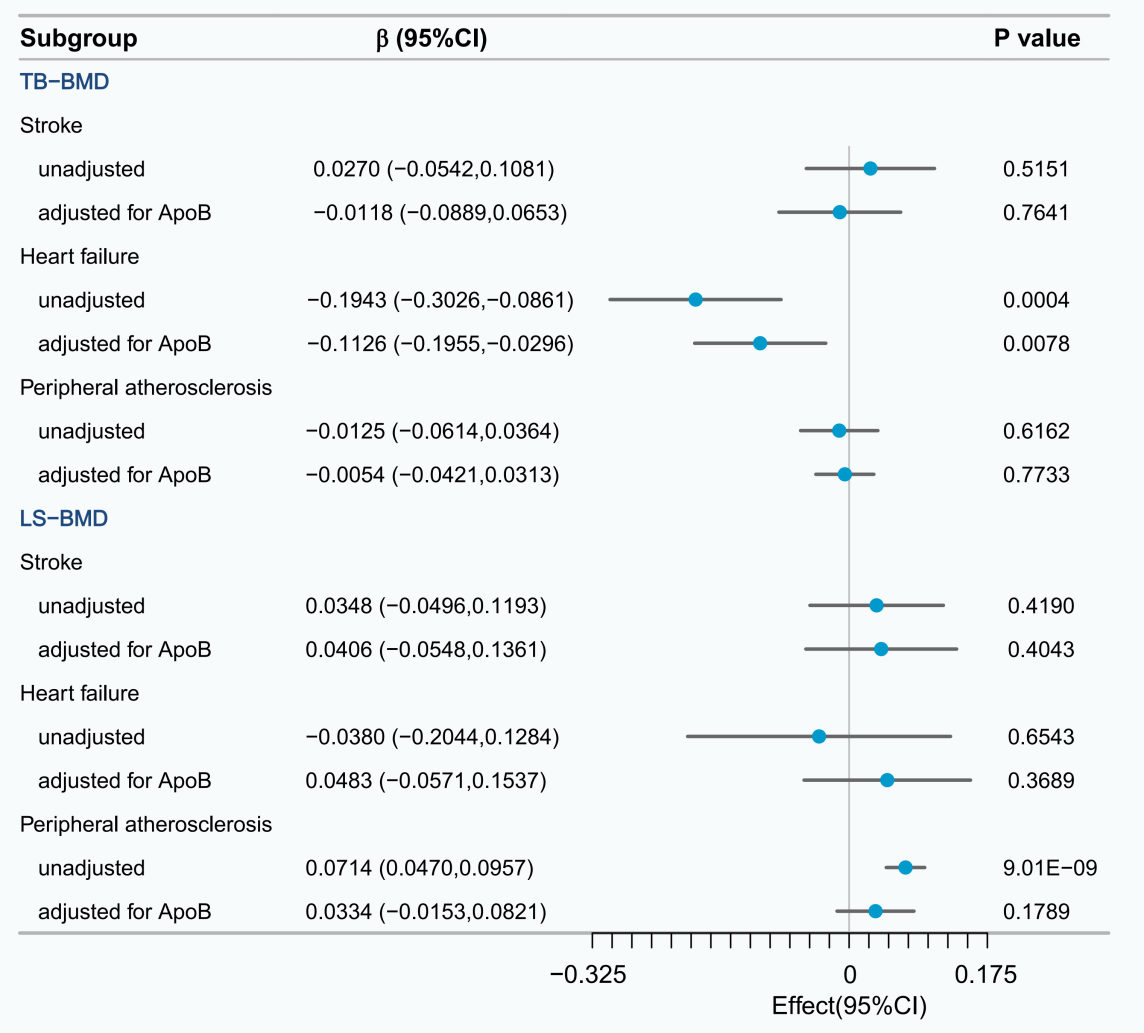
**UVMR and multivariable mendelian randomization (MVMR) analysis of the effect of potential mediator on TB-BMD and LS-BMD**.

The causal relationship between heart failure and TB-BMD remained regardless of ApoB adjustment (β_M⁢V⁢M⁢R_ = –0.1126, 95% CI: –0.1955 to –0.0296, *p* = 0.0078) (Fig. 4). Sensitivity analysis indicated that there was no heterogeneity or horizontal pleiotropy in the causal relationship between heart failure and TB-BMD (**Supplementary Table 12**). Based on the following screening process, we ultimately confirmed that heart failure can mediate the causal relationship between ApoB and TB-BMD. (1) There should be a unidirectional causal relationship between ApoB and TB-BMD. (2) A causal relationship between heart failure and TB-BMD should still exist regardless of whether ApoB is adjusted. (3) The relationship between ApoB and heart failure should be in the same direction as the relationship between heart failure and TB-BMD. We applied a two-step MR analysis to assess the mediating effect of heart failure on the causal relationship between ApoB and TB-BMD. Heart failure explained 18.69% (2.18%, 35.21%) of the total effect of serum ApoB levels on TB-BMD (*p* < 0.05). No causal relationships were identified between other cardiovascular diseases and either TB-BMD or LS-BMD.

## 4. Discussion

This study investigated the association and potential causal relationship between ApoB with TB-BMD and LS-BMD, as well as whether cardiovascular diseases could mediate this causal relationship. We initially conducted an epidemiological observational analysis using the NHANES database. The results indicated that, after adjusting for various confounding factors, there was a negative correlation between serum ApoB with TB-BMD and LS-BMD. Further analysis revealed that this relationship was linear. The MR analysis further demonstrated a causal relationship between ApoB with TB-BMD and LS-BMD. Furthermore, we identified heart failure as a mediating factor in the causal relationship between ApoB and TB-BMD, with a mediation proportion of 18.69%.

Previous research has highlighted a correlation between lipid and bone metabolism, via intricate underlying mechanisms [34]. Bone marrow stromal cells require physiologic levels of cholesterol synthesis to undergo the process of osteogenic differentiation [35], while exogenous cholesterol serves as an inhibitor of this differentiation [36]. Cholesterol plays a role in bone metabolism through one of its essential byproducts, vitamin D, which is crucial for maintaining bone calcification [37]. Previous research has observed a negative correlation between serum total cholesterol levels and 25-hydroxyvitamin D (25(OH)D) levels [38]. Even when accounting for age and BMI adjustments, individuals with familial hypercholesterolemia may still encounter a reduction in BMD [39]. While elevated cholesterol levels can lead to arterial calcification, they often exhibit an opposing effect by reducing calcification in bones [40]. The influence of circulating lipids on bone metabolism has been extensively researched. However, studies focusing on ApoB have been relatively scarce. The protein component of lipoproteins, known as apolipoprotein, plays a crucial role in lipoprotein metabolism and has important physiological functions [41]. The liver synthesizes ApoB, which serves as the principal structural protein within low-density lipoprotein (LDL) [42]. ApoB provides an accurate measurement of both very low-density lipoprotein (VLDL) and LDL particle counts, making it a dependable surrogate for the actual count of LDL particles [43]. Therefore, ApoB may also be a valuable indicator for assessing its independent impact on bone health, separate from other lipids.

The evidence directly associating ApoB with bone health is limited. A study from NHANES showed that serum ApoB negatively correlates with lumbar spine BMD in females, which is similar to our findings [7]. Recent studies have shown that ApoB, unaffected by age, demonstrates higher sensitivity and specificity in predicting cardiovascular events. An MR study has indicated that ApoB is a key factor influencing the occurrence of coronary heart disease, distinct from cholesterol and triglycerides [44]. Our MR analysis also confirmed a causal relationship between ApoB and cardiovascular diseases, including stroke, coronary artery disease, heart failure, and peripheral atherosclerosis. An increasing volume of evidence highlights the interconnection between diseases of the vascular system and bone metabolism. Some scholars have introduced the concept of a bone-vascular axis, suggesting the existence of bidirectional endocrine and metabolic signals between the vascular system and bones. Disruptions in the bone-vascular axis, brought about by metabolic abnormalities, may lead to the simultaneous occurrence of vascular and skeletal disorders [45]. Previous studies support this concept, as elderly individuals with osteoporosis often exhibit a higher risk of cardiovascular disease compared to those without osteoporosis [46, 47]. Interestingly, despite increasing evidence of the association between BMD and cardiovascular disease, a prospective cohort study found a lack of clear association between BMD and the risk of cardiovascular disease. Through studying its experimental strategy, we found that this may be related to the shorter follow-up time. Additionally, the study measured the BMD of the non-dominant arm, while our research often focuses on the BMD of the lumbar spine and femur, which may also be a contributing factor. Further in-depth research is needed in this area in the future [12]. In addition, lipid-lowering medications may play a role in preserving BMD and reducing the risk of osteoporotic fractures [48, 49], while high-fat diets have been associated with decreased BMD in animal studies [50]. Some researchers have suggested a potential link between osteoporosis and atherosclerosis [51]. Our MR study found a positive causal relationship between the occurrence of peripheral atherosclerosis and bone density, which disappeared after adjusting for ApoB. Some studies have also reported a positive correlation between the lipid profiles associated with atherosclerosis and BMD [52]. This might be due to the similarity observed between the calcification process of atherosclerosis and processes observed in bone remodeling, as well as the existence of shared regulatory factors [53].

Our study also confirmed the mediating role of heart failure and quantified its proportion in the causal relationship between ApoB and TB-BMD. Although lipoproteins are typically not viewed as the primary risk factor for heart failure, certain studies suggest an association with the occurrence of heart failure [54, 55]. A study in Sweden showed a positive link between ApoB/ApoA-1 levels and the onset of heart failure [56]. Furthermore, a recent MR analysis proposed that therapies aimed at lowering lipids related to ApoB might provide greater advantages in lowering the risk of heart failure [57]. There is considerable research supporting the association between heart failure and a heightened risk of low BMD [58]. Shared risk factors between the two include reduced exercise endurance, decreased levels of 25(OH)D, and the presence of diabetes mellitus. The reduced physical activity in heart failure patients may impact their vitamin D levels and subsequently affect bone metabolism [59]. Heart failure patients elevate their adiponectin levels internally to counteract metabolic damage caused by the disease, and adiponectin has been confirmed as one of the lipid factors most associated with decreased BMD [60]. Furthermore, they exhibit common pathogenic mechanisms, such as activation of the renin-angiotensin-aldosterone system, elevated levels of parathyroid hormone, and the response to oxidative stress [61]. In future mechanistic studies, we can further explore the mechanisms by which ApoB directly affects bone cells (osteoblasts/osteoclasts) through lipoprotein particles, for example, through oxidative stress or inflammatory signals. At the same time, we can also analyze the driving factors for the reduction of BMD in heart failure patients, such as insufficient bone perfusion due to decreased cardiac output or neurohormonal activation [62].

This study stands as the first comprehensive exploration delving into the relationship between serum ApoB and BMD using an epidemiological observational analysis with population-wide data and MR studies of large-scale genetic data. These findings support the theory that elevated serum levels of ApoB adversely affect bone health and suggests that improving heart function might be an important target for intervening in osteoporosis associated with elevated ApoB levels. These findings are significant for assessing the long-term effects of ApoB-lowering drugs on BMD and the bone protective effects in high-risk heart failure populations, and they provide important guidance on whether early intervention in heart failure is crucial in reversing the impact of elevated ApoB on BMD.

This study has several strengths. Firstly, in our observational study, we conducted adjustments for a multitude of confounding factors closely associated with BMD and had a relatively large sample size. Secondly, we established strict mediation screening criteria to ensure the credibility of the results. Additionally, consistent outcomes were observed across various sensitivity analyses, reflecting the robustness of the evidence and suggesting that confounding factors are unlikely to explain the observed correlations. Nevertheless, there are certain limitations present in this study. First, despite our use of various MR methods to mitigate pleiotropy-related confusion, it is important to acknowledge the presence of residual bias, a recognized limitation of MR studies. Second, MR analysis typically examines the lifelong influence of risk factors on outcomes, which can complicate the identification of causal effects at various stages of disease development. Third, our observational study and genetic data were obtained from different populations, with the cross-sectional study conducted among the American population and the MR study involving individuals of European ancestry, this may lead to results that cannot be generalized to other races or regions. Fourth, the age range of the study subjects is 20–59 years, which does not include the elderly population that is at high risk for osteoporosis, limiting the generalizability of the conclusions. At the same time, we also lack a discussion on the potential impact of different age stages on BMD, such as the fact that bone loss is often more common in postmenopausal women. In the future, we could additionally include different age groups to eliminate such confounding factors. Regarding data sources, there may be differences in measurement standards of different GWAS data, lifestyle habits of populations, and methods of covariate adjustment, which could affect the consistency of results. Additionally, the GWAS data for candidate mediating diseases may come from different sources, and their diagnostic criteria or phenotype definitions may not be consistent. Furthermore, in terms of statistical methods, although we tested for horizontal pleiotropy using methods like MR-Egger, we cannot completely rule out the potential bias of instrumental variables affecting the results through other unknown pathways. We also simplified the mediation analysis by only quantifying the mediating proportion of heart failure, without exploring the effects of other potential mediating factors such as inflammatory markers and vitamin D levels. Given these limitations, this finding needs to be validated in well-designed prospective cohort studies and cross-ethnic cohort studies, combined with metabolomics and proteomics data, to identify key biomarkers in the ApoB-BMD association and further investigate its potential mechanisms.

## 5. Conclusions

Through cross-sectional study and MR analysis, we elucidated the association between elevated serum ApoB levels and decreased TB-BMD and LS-BMD. Furthermore, we discovered that heart failure may mediate the causal relationship between ApoB and TB-BMD. The study findings indicated that reducing serum ApoB levels could improve BMD. In addition, in individuals with suboptimal ApoB control, preventing heart failure may have mitigated the detrimental effects of decreased BMD resulting from elevated ApoB levels.

## Availability of Data and Materials

Publicly available datasets are available online for this study. The NHANES repository/repositories name and accession numbers are available online at (https://www.cdc.gov/nchs/nhanes/index.html). The entirety of the data used in this MR study was sourced from the publicly accessible Open GWAS project database (https://gwas.mrcieu.ac.uk/), available for free to all interested parties. Parts of the graphical abstract were drawn by using pictures from Servier Medical Art. Servier Medical Art by Servier is licensed under a Creative Commons Attribution 3.0 Unported License (https://creativecommons.org/licenses/by/3.0/). All information used for this study is publicly available as deidentified GWAS summary statistics.

## Supplementary Material

Supplementary material associated with this article can be found, in the online version, at https://doi.org/10.31083/RCM31395.

## References

[1] Sànchez-Riera L, Carnahan E, Vos T, Veerman L, Norman R, Lim SS (2014). The global burden attributable to low bone mineral density. *Annals of the Rheumatic Diseases*.

[2] Castaneda M, Strong JM, Alabi DA, Hernandez CJ (2020). The Gut Microbiome and Bone Strength. *Current Osteoporosis Reports*.

[3] Johnell O, Kanis JA, Oden A, Johansson H, De Laet C, Delmas P (2005). Predictive value of BMD for hip and other fractures. *Journal of Bone and Mineral Research: the Official Journal of the American Society for Bone and Mineral Research*.

[4] Burge R, Dawson-Hughes B, Solomon DH, Wong JB, King A, Tosteson A (2007). Incidence and economic burden of osteoporosis-related fractures in the United States, 2005-2025. *Journal of Bone and Mineral Research: the Official Journal of the American Society for Bone and Mineral Research*.

[5] Anagnostis P, Florentin M, Livadas S, Lambrinoudaki I, Goulis DG (2022). Bone Health in Patients with Dyslipidemias: An Underestimated Aspect. *International Journal of Molecular Sciences*.

[6] Bagger YZ, Rasmussen HB, Alexandersen P, Werge T, Christiansen C, Tankó LB (2007). Links between cardiovascular disease and osteoporosis in postmenopausal women: serum lipids or atherosclerosis per se?. *Osteoporosis International: a Journal Established as Result of Cooperation between the European Foundation for Osteoporosis and the National Osteoporosis Foundation of the USA*.

[7] Zhu R, Xu Y, Wang Z, Li H, Song M, Wan H (2022). Higher serum apolipoprotein B level will reduce the bone mineral density and increase the risk of osteopenia or osteoporosis in adults. *Frontiers in Cell and Developmental Biology*.

[8] Ference BA, Ginsberg HN, Graham I, Ray KK, Packard CJ, Bruckert E (2017). Low-density lipoproteins cause atherosclerotic cardiovascular disease. 1. Evidence from genetic, epidemiologic, and clinical studies. A consensus statement from the European Atherosclerosis Society Consensus Panel. *European Heart Journal*.

[9] Kan B, Zhao Q, Wang L, Xue S, Cai H, Yang S (2021). Association between lipid biomarkers and osteoporosis: a cross-sectional study. *BMC Musculoskeletal Disorders*.

[10] Sun X, Wu X (2023). Association of apolipoprotein A1 with osteoporosis: a cross-sectional study. *BMC Musculoskeletal Disorders*.

[11] Yang Y, Huang Y (2023). Association between bone mineral density and cardiovascular disease in older adults. *Frontiers in Public Health*.

[12] Bhatta L, Cepelis A, Vikjord SA, Malmo V, Laugsand LE, Dalen H (2021). Bone mineral density and risk of cardiovascular disease in men and women: the HUNT study. *European Journal of Epidemiology*.

[13] Farhat GN, Cauley JA (2008). The link between osteoporosis and cardiovascular disease. Clinical Cases in Mineral and Bone Metabolism: the Official Journal of the Italian Society of Osteoporosis, Mineral Metabolism, and Skeletal Diseases. *Clinical Cases in Mineral and Bone Metabolism: the Official Journal of the Italian Society of Osteoporosis, Mineral Metabolism, and Skeletal Diseases*.

[14] Pan XB, Ma QY, Gao T, Zhang T, Xun J, Ma XT (2025). Osteoporosis risk and its association with all-cause and cause-specific mortality among the elderly: a 16-year nationwide cohort study. *BMC Geriatrics*.

[15] Domiciano DS, Machado LG, Lopes JB, Figueiredo CP, Caparbo VF, Oliveira RM (2016). Bone Mineral Density and Parathyroid Hormone as Independent Risk Factors for Mortality in Community-Dwelling Older Adults: A Population-Based Prospective Cohort Study in Brazil. The São Paulo Ageing & Health (SPAH) Study. *Journal of Bone and Mineral Research: the Official Journal of the American Society for Bone and Mineral Research*.

[16] Roth GA, Mensah GA, Johnson CO, Addolorato G, Ammirati E, Baddour LM (2020). Global Burden of Cardiovascular Diseases and Risk Factors, 1990-2019: Update From the GBD 2019 Study. *Journal of the American College of Cardiology*.

[17] Qiao J, Zhang M, Wang T, Huang S, Zeng P (2021). Evaluating Causal Relationship Between Metabolites and Six Cardiovascular Diseases Based on GWAS Summary Statistics. *Frontiers in Genetics*.

[18] Smith GD, Timpson N, Ebrahim S (2008). Strengthening causal inference in cardiovascular epidemiology through Mendelian randomization. *Annals of Medicine*.

[19] Zipf G, Chiappa M, Porter KS, Ostchega Y, Lewis BG, Dostal J (2013). National health and nutrition examination survey: plan and operations, 1999-2010. *Vital and Health Statistics. Ser. 1, Programs and Collection Procedures*.

[20] Wang J, Xing F, Sheng N, Xiang Z (2022). Associations of the Geriatric Nutritional Risk Index With Femur Bone Mineral Density and Osteoporosis in American Postmenopausal Women: Data From the National Health and Nutrition Examination Survey. *Frontiers in Nutrition*.

[21] Wang G, Fang ZB, Liu DL, Chu SF, Li HL, Zhao HX (2022). Association between caffeine intake and lumbar spine bone mineral density in adults aged 20-49: A cross-sectional study. *Frontiers in Endocrinology*.

[22] Barton AR, Sherman MA, Mukamel RE, Loh PR (2021). Whole-exome imputation within UK Biobank powers rare coding variant association and fine-mapping analyses. *Nature Genetics*.

[23] Medina-Gomez C, Kemp JP, Trajanoska K, Luan J, Chesi A, Ahluwalia TS (2018). Life-Course Genome-wide Association Study Meta-analysis of Total Body BMD and Assessment of Age-Specific Effects. *American Journal of Human Genetics*.

[24] Li GHY, Cheung CL, Au PCM, Tan KCB, Wong ICK, Sham PC (2020). Positive effects of low LDL-C and statins on bone mineral density: an integrated epidemiological observation analysis and Mendelian randomization study. *International Journal of Epidemiology*.

[25] Zheng HF, Forgetta V, Hsu YH, Estrada K, Rosello-Diez A, Leo PJ (2015). Whole-genome sequencing identifies EN1 as a determinant of bone density and fracture. *Nature*.

[26] Burgess S, Scott RA, Timpson NJ, Davey Smith G, Thompson SG, EPIC- InterAct Consortium (2015). Using published data in Mendelian randomization: a blueprint for efficient identification of causal risk factors. *European Journal of Epidemiology*.

[27] Wu F, Huang Y, Hu J, Shao Z (2020). Mendelian randomization study of inflammatory bowel disease and bone mineral density. *BMC Medicine*.

[28] Carter AR, Sanderson E, Hammerton G, Richmond RC, Davey Smith G, Heron J (2021). Mendelian randomisation for mediation analysis: current methods and challenges for implementation. *European Journal of Epidemiology*.

[29] Bowden J, Davey Smith G, Burgess S (2015). Mendelian randomization with invalid instruments: effect estimation and bias detection through Egger regression. *International Journal of Epidemiology*.

[30] Chen Z, Chen Z, Jin X (2023). Mendelian randomization supports causality between overweight status and accelerated aging. *Aging Cell*.

[31] Bowden J, Davey Smith G, Haycock PC, Burgess S (2016). Consistent Estimation in Mendelian Randomization with Some Invalid Instruments Using a Weighted Median Estimator. *Genetic Epidemiology*.

[32] Verbanck M, Chen CY, Neale B, Do R (2018). Publisher Correction: Detection of widespread horizontal pleiotropy in causal relationships inferred from Mendelian randomization between complex traits and diseases. *Nature Genetics*.

[33] Glickman ME, Rao SR, Schultz MR (2014). False discovery rate control is a recommended alternative to Bonferroni-type adjustments in health studies. *Journal of Clinical Epidemiology*.

[34] Mandal CC (2015). High Cholesterol Deteriorates Bone Health: New Insights into Molecular Mechanisms. *Frontiers in Endocrinology*.

[35] Parhami F, Mody N, Gharavi N, Ballard AJ, Tintut Y, Demer LL (2002). Role of the cholesterol biosynthetic pathway in osteoblastic differentiation of marrow stromal cells. *Journal of Bone and Mineral Research: the Official Journal of the American Society for Bone and Mineral Research*.

[36] Li K, Xiu C, Zhou Q, Ni L, Du J, Gong T (2019). A dual role of cholesterol in osteogenic differentiation of bone marrow stromal cells. *Journal of Cellular Physiology*.

[37] Song Y, Liu J, Zhao K, Gao L, Zhao J (2021). Cholesterol-induced toxicity: An integrated view of the role of cholesterol in multiple diseases. *Cell Metabolism*.

[38] Cutillas-Marco E, Prosper AF, Grant WB, Morales-Suárez-Varela MM (2013). Vitamin D status and hypercholesterolemia in Spanish general population. *Dermato-endocrinology*.

[39] Yerges-Armstrong LM, Shen H, Ryan KA, Streeten EA, Shuldiner AR, Mitchell BD (2013). Decreased bone mineral density in subjects carrying familial defective apolipoprotein B-100. *The Journal of Clinical Endocrinology and Metabolism*.

[40] Awan Z, Alwaili K, Alshahrani A, Langsetmo L, Goltzman D, Genest J (2010). Calcium homeostasis and skeletal integrity in individuals with familial hypercholesterolemia and aortic calcification. *Clinical Chemistry*.

[41] Florea G, Tudorache IF, Fuior EV, Ionita R, Dumitrescu M, Fenyo IM (2022). Apolipoprotein A-II, a Player in Multiple Processes and Diseases. *Biomedicines*.

[42] Bell DA, Hooper AJ, Burnett JR (2011). Mipomersen, an antisense apolipoprotein B synthesis inhibitor. *Expert Opinion on Investigational Drugs*.

[43] Sniderman AD, Thanassoulis G, Glavinovic T, Navar AM, Pencina M, Catapano A (2019). Apolipoprotein B Particles and Cardiovascular Disease: A Narrative Review. *JAMA Cardiology*.

[44] Richardson TG, Sanderson E, Palmer TM, Ala-Korpela M, Ference BA, Davey Smith G (2020). Evaluating the relationship between circulating lipoprotein lipids and apolipoproteins with risk of coronary heart disease: A multivariable Mendelian randomisation analysis. *PLoS Medicine*.

[45] Thompson B, Towler DA (2012). Arterial calcification and bone physiology: role of the bone-vascular axis. *Nature Reviews. Endocrinology*.

[46] Samelson EJ, Kiel DP, Broe KE, Zhang Y, Cupples LA, Hannan MT (2004). Metacarpal cortical area and risk of coronary heart disease: the Framingham Study. *American Journal of Epidemiology*.

[47] Varosy PD, Shlipak MG, Vittinghoff E, Black DM, Herrington D, Hulley SB (2003). Fracture and the risk of coronary events in women with heart disease. *The American Journal of Medicine*.

[48] Wang PS, Solomon DH, Mogun H, Avorn J (2000). HMG-CoA reductase inhibitors and the risk of hip fractures in elderly patients. *JAMA*.

[49] Chan KA, Andrade SE, Boles M, Buist DS, Chase GA, Donahue JG (2000). Inhibitors of hydroxymethylglutaryl-coenzyme A reductase and risk of fracture among older women. *Lancet (London, England)*.

[50] Parhami F, Tintut Y, Beamer WG, Gharavi N, Goodman W, Demer LL (2001). Atherogenic high-fat diet reduces bone mineralization in mice. *Journal of Bone and Mineral Research: the Official Journal of the American Society for Bone and Mineral Research*.

[51] Jeong IK, Cho SW, Kim SW, Choi HJ, Park KS, Kim SY (2010). Lipid profiles and bone mineral density in pre- and postmenopausal women in Korea. *Calcified Tissue International*.

[52] Adami S, Braga V, Zamboni M, Gatti D, Rossini M, Bakri J (2004). Relationship between lipids and bone mass in 2 cohorts of healthy women and men. *Calcified Tissue International*.

[53] Azeez TA (2023). Osteoporosis and cardiovascular disease: a review. *Molecular Biology Reports*.

[54] Holme I, Strandberg TE, Faergeman O, Kastelein JJP, Olsson AG, Tikkanen MJ (2009). Congestive heart failure is associated with lipoprotein components in statin-treated patients with coronary heart disease Insights from the Incremental Decrease in End points Through Aggressive Lipid Lowering Trial (IDEAL). *Atherosclerosis*.

[55] Dhingra R, Sesso HD, Kenchaiah S, Gaziano JM (2008). Differential effects of lipids on the risk of heart failure and coronary heart disease: the Physicians’ Health Study. *American Heart Journal*.

[56] Ingelsson E, Arnlöv J, Sundström J, Zethelius B, Vessby B, Lind L (2005). Novel metabolic risk factors for heart failure. *Journal of the American College of Cardiology*.

[57] Dai H, Hou T, Wang Q, Hou Y, Wang T, Zheng J (2023). Causal relationships between the gut microbiome, blood lipids, and heart failure: a Mendelian randomization analysis. *European Journal of Preventive Cardiology*.

[58] Aluoch AO, Jessee R, Habal H, Garcia-Rosell M, Shah R, Reed G (2012). Heart failure as a risk factor for osteoporosis and fractures. *Current Osteoporosis Reports*.

[59] Gua C, Li T, Wang J (2022). Causal association between heart failure and bone mineral density: Insights from a two-sample bidirectional Mendelian randomization study. *Genomics*.

[60] Barbour KE, Zmuda JM, Boudreau R, Strotmeyer ES, Horwitz MJ, Evans RW (2011). Adipokines and the risk of fracture in older adults. *Journal of Bone and Mineral Research: the Official Journal of the American Society for Bone and Mineral Research*.

[61] Loncar G, Cvetinovic N, Lainscak M, Isaković A, von Haehling S (2020). Bone in heart failure. *Journal of Cachexia, Sarcopenia and Muscle*.

[62] Xing W, Lv X, Gao W, Wang J, Yang Z, Wang S (2018). Bone mineral density in patients with chronic heart failure: a meta-analysis. *Clinical Interventions in Aging*.

