# Supplementary figures and images for: Association Between Serum Apolipoprotein B and Bone Mineral Density and the Effects of Cardiovascular Disease Mediation: Results From the NHANES 2011–2016 and a Mendelian Randomization Study
Source: Rev Cardiovasc Med. 2025 May 23;26(5):31395. doi: 10.31083/RCM31395 (PMC12135671; doi:10.31083/RCM31395)

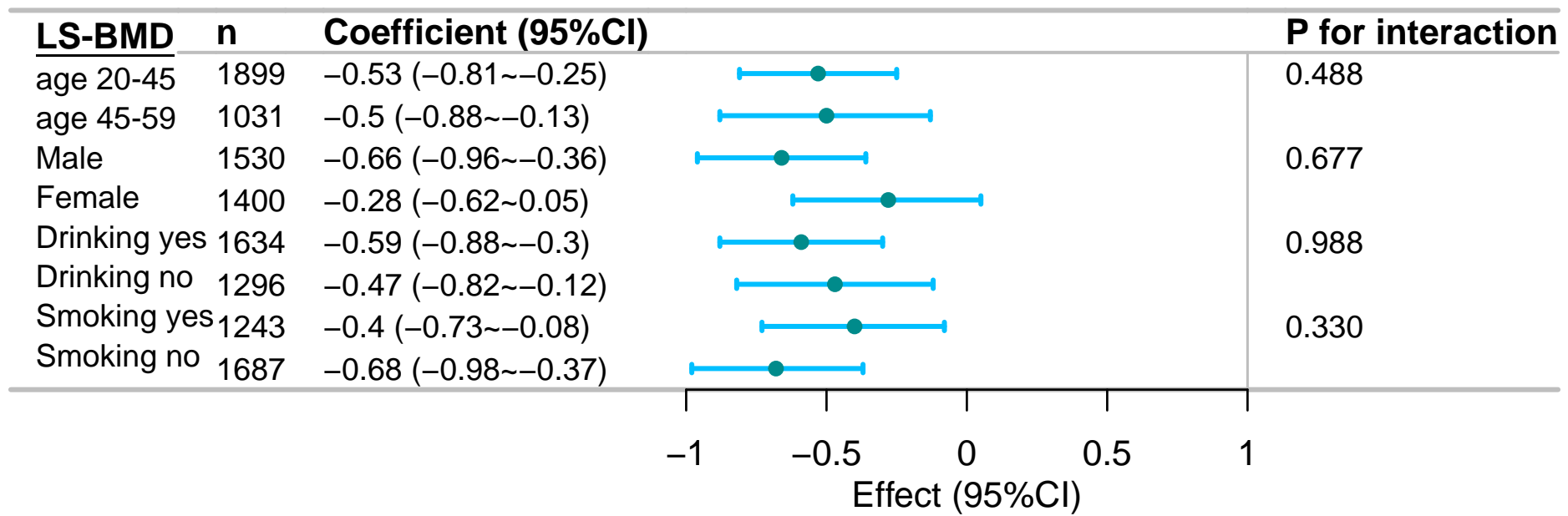

Supplement: Supplementary file 1 [file 2153-8174-26-5-31395-s1.zip › Supplementary Fig. 1.pdf]

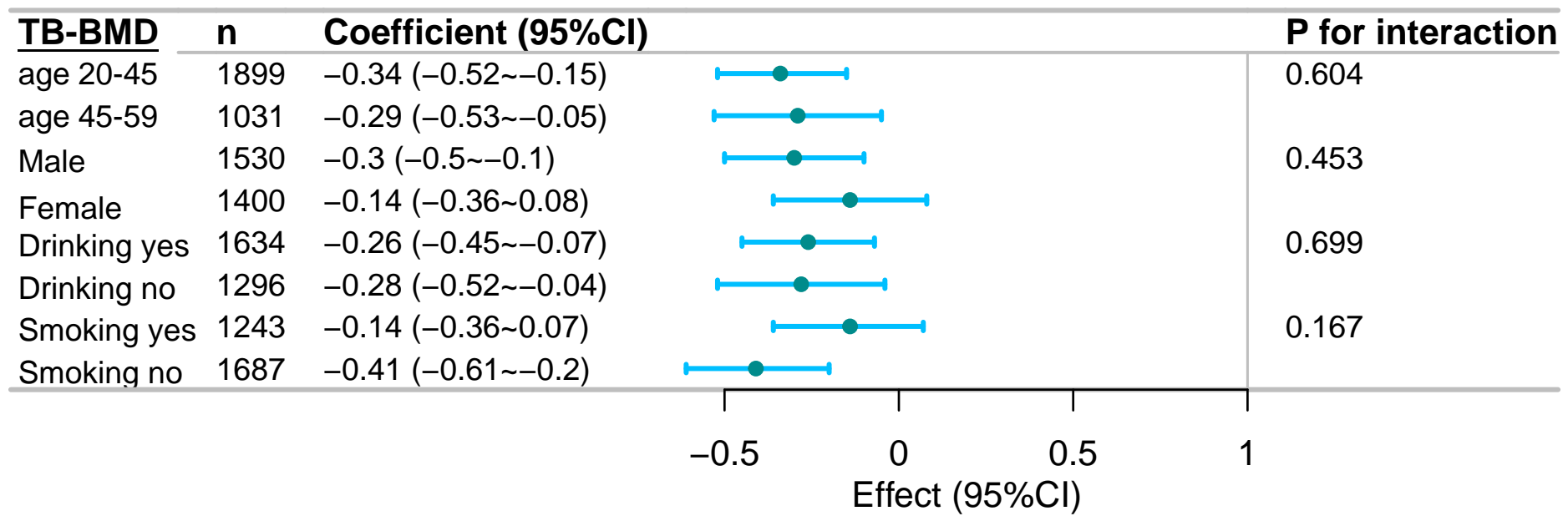

Supplement: Supplementary file 1 [file 2153-8174-26-5-31395-s1.zip › Supplementary Fig. 2.pdf]
