# Supplementary material for: Association Between Serum Apolipoprotein B and Bone Mineral Density and the Effects of Cardiovascular Disease Mediation: Results From the NHANES 2011–2016 and a Mendelian Randomization Study
Source: Rev Cardiovasc Med. 2025 May 23;26(5):31395. doi: 10.31083/RCM31395 (PMC12135671; doi:10.31083/RCM31395)
Supplement: Supplementary file 1 [file 2153-8174-26-5-31395-s1.zip › Supplementary Table 1.doc]

**Table S1.** Information on mediator data sources.

| **Consortium** | **Author** | **Phenotype** | **Population** | **Cases** | **Controls** | **PMID** |
| --- | --- | --- | --- | --- | --- | --- |
| MEGASTROKE | Malik R et al. | Stroke | European | 40,585 | 406,111 | 29531354 |
| UKBB | van der Harst P et al. | Coronary artery disease | European | 122,733 | 424,528 | 29212778 |
| CSG | Nielsen JB et al. | Atrial fibrillation | European | 60,620 | 970,216 | 30061737 |
| HERMES | Shah S et al. | Heart failure | European | 47,309 | 930,014 | 31919418 |
| The FinnGen consortium | ... | Venous thromboembolism | European | 9,176 | 209,616 | ... |
| The FinnGen consortium | ... | Peripheral atherosclerosis | European | 6,631 | 162,201 | ... |

UKBB, The UK Biobank study; CSG, Center for Statistical Genetics; HERMES, Heart Failure Molecular Epidemiology for Therapeutic Targets.
