# Supplementary material for: Association Between Serum Apolipoprotein B and Bone Mineral Density and the Effects of Cardiovascular Disease Mediation: Results From the NHANES 2011–2016 and a Mendelian Randomization Study
Source: Rev Cardiovasc Med. 2025 May 23;26(5):31395. doi: 10.31083/RCM31395 (PMC12135671; doi:10.31083/RCM31395)
Supplement: Supplementary file 1 [file 2153-8174-26-5-31395-s1.zip › Supplementary Table 10.doc]

**Table S10.** Reverse association analyses using IVW method.

|  | β | SE | P-value |
| --- | --- | --- | --- |
| TB-BMD on ApoB | 0.0026 | 0.0112 | 0.8142 |
| LS-BMD on ApoB | -0.0042 | 0.0099 | 0.6730 |
| Stroke on ApoB | -0.0001 | 0.0149 | 0.9929 |
| Coronary artery disease on ApoB | -0.0383 | 0.0155 | 0.0132 |
| Heart failure on ApoB | -0.0308 | 0.0615 | 0.6166 |
| Peripheral atherosclerosis | 0.1833 | 0.2123 | 0.3878 |
