# Supplementary material for: Association Between Serum Apolipoprotein B and Bone Mineral Density and the Effects of Cardiovascular Disease Mediation: Results From the NHANES 2011–2016 and a Mendelian Randomization Study
Source: Rev Cardiovasc Med. 2025 May 23;26(5):31395. doi: 10.31083/RCM31395 (PMC12135671; doi:10.31083/RCM31395)
Supplement: Supplementary file 1 [file 2153-8174-26-5-31395-s1.zip › Supplementary Table 11.doc]

**Table S11**. Sensitivity analysis of serum ApoB on cardiovascular diseases.

|  | Heterogeneity test | | | |  | Pleiotropy test | | | |
| --- | --- | --- | --- | --- | --- | --- | --- | --- | --- |
|  | Cochrane's Q test | |  | |  | MR-Egger intercept test | |  | |
| apolipoprotein B on | Q | P |  |  |  | Intercept | P |  |  |
| Stroke | 411.980 | 1.346e-20 |  |  |  | -0.002 | 0.197 |  |  |
| Coronary artery disease | 387.254 | 4.186e-23 |  |  |  | -0.002 | 0.198 |  |  |
| Atrial fibrillation | 430.580 | 5.284e-22 |  |  |  | -0.002 | 0.112 |  |  |
| Heart failure | 347.273 | 1.485e-15 |  |  |  | 0.000 | 0.850 |  |  |
| Venous thromboembolism | 286.346 | 9.773e-07 |  |  |  | 0.002 | 0.373 |  |  |
| Peripheral atherosclerosis | 316.206 | 1.502e-09 |  |  |  | -0.000 | 0.836 |  |  |
