# Supplementary material for: Association Between Serum Apolipoprotein B and Bone Mineral Density and the Effects of Cardiovascular Disease Mediation: Results From the NHANES 2011–2016 and a Mendelian Randomization Study
Source: Rev Cardiovasc Med. 2025 May 23;26(5):31395. doi: 10.31083/RCM31395 (PMC12135671; doi:10.31083/RCM31395)
Supplement: Supplementary file 1 [file 2153-8174-26-5-31395-s1.zip › Supplementary Table 12.doc]

**Table S12.** Sensitivity analysis of candidate mediators on TB-BMD and LS-BMD.

|  | Heterogeneity test | | | |  | Pleiotropy test | | | |
| --- | --- | --- | --- | --- | --- | --- | --- | --- | --- |
|  | Cochrane's Q test | |  | |  | MR-Egger intercept test | |  | |
| TB-BMD | Q | P |  |  |  | Intercept | P |  |  |
| Stroke | 37.160 | 0.002 |  |  |  | 0.000 | 0.978 |  |  |
| Heart failure | 6.531 | 0.163 |  |  |  | -0.015 | 0.296 |  |  |
| Peripheral atherosclerosis | 9.662 | 0.085 |  |  |  | 0.009 | 0.684 |  |  |
| LS-BMD |  |  |  |  |  |  |  |  |  |
| Stroke | 23.710 | 0.096 |  |  |  | 0.019 | 0.094 |  |  |
| Heart failure | 8.247 | 0.083 |  |  |  | -0.013 | 0.620 |  |  |
| Peripheral atherosclerosis | 1.067 | 0.957 |  |  |  | 0.012 | 0.587 |  |  |
