# Supplementary material for: Association Between Serum Apolipoprotein B and Bone Mineral Density and the Effects of Cardiovascular Disease Mediation: Results From the NHANES 2011–2016 and a Mendelian Randomization Study
Source: Rev Cardiovasc Med. 2025 May 23;26(5):31395. doi: 10.31083/RCM31395 (PMC12135671; doi:10.31083/RCM31395)
Supplement: Supplementary file 1 [file 2153-8174-26-5-31395-s1.zip › Supplementary Table 2.doc]

**Table S2.** The F statistics for each instrument-exposure association in MR study.

|  | F statistics range |
| --- | --- |
| ApoB on TB-BMD | 20.73-29776.66 |
| ApoB on LS-BMD | 20.73-4513.33 |
| ApoB on Stroke | 20.73-29776.66 |
| Apob on Coronary artery disease | 20.73-2761.19 |
| Apob on Atrial fibrillation | 20.73-29776.66 |
| Apob on Heart failure | 20.73-29776.66 |
| Apob on Venous thromboembolism | 20.73-29776.66 |
| Apob on Peripheral atherosclerosis | 20.73-29776.66 |

The F statistic was defined by the formula: F = R2/(1-R2) × (N-k-1)/k. Here, R2 represented the proportion of exposure variance explained by the IVs, N denoted the sample size of exposure, and k represented the count of IVs.
