# Supplementary material for: Association Between Serum Apolipoprotein B and Bone Mineral Density and the Effects of Cardiovascular Disease Mediation: Results From the NHANES 2011–2016 and a Mendelian Randomization Study
Source: Rev Cardiovasc Med. 2025 May 23;26(5):31395. doi: 10.31083/RCM31395 (PMC12135671; doi:10.31083/RCM31395)
Supplement: Supplementary file 1 [file 2153-8174-26-5-31395-s1.zip › Supplementary Table 3.doc]

**Table S3.** Baseline characteristics of the study population.

| **Variables** | **Apolipoprotein B** | | | | | **P-value** |
| --- | --- | --- | --- | --- | --- | --- |
|  | **Total**  **(n = 2,930)** | **Q1**  **(n = 702)** | **Q2**  **(n = 756)** | **Q3**  **(n = 723)** | **Q4**  **(n = 749)** |  |
| Gender, n (%) |  |  |  |  |  | < 0.001 |
| Male | 1530 (52.2) | 311 (44.3) | 374 (49.5) | 380 (52.6) | 465 (62.1) |  |
| Female | 1400 (47.8) | 391 (55.7) | 382 (50.5) | 343 (47.4) | 284 (37.9) |  |
| Age, (years) | 38.9 ± 11.5 | 34.1 ± 11.4 | 37.6 ± 11.7 | 40.7 ± 11.0 | 43.0 ± 10.1 | < 0.001 |
| Race, n (%) |  |  |  |  |  | < 0.001 |
| Mexican American | 425 (14.5) | 73 (10.4) | 97 (12.8) | 122 (16.9) | 133 (17.8) |  |
| Other Hispanic | 312 (10.6) | 62 (8.8) | 69 (9.1) | 81 (11.2) | 100 (13.4) |  |
| Non-Hispanic White | 1145 (39.1) | 260 (37) | 314 (41.5) | 281 (38.9) | 290 (38.7) |  |
| Non-Hispanic Black | 574 (19.6) | 174 (24.8) | 152 (20.1) | 124 (17.2) | 124 (16.6) |  |
| Other Race | 474 (16.2) | 133 (18.9) | 124 (16.4) | 115 (15.9) | 102 (13.6) |  |
| Education level, n (%) |  |  |  |  |  | < 0.001 |
| Did not graduate from high school | 531 (17.5) | 99 (13.1) | 128 (17) | 135 (18.1) | 169 (21.8) |  |
| Graduated from high school | 659 (21.8) | 140 (18.5) | 163 (21.7) | 152 (20.4) | 204 (26.3) |  |
| College education or above | 1836 (60.7) | 516 (68.3) | 460 (61.3) | 458 (61.5) | 402 (51.9) |  |
| Martial status, n (%) |  |  |  |  |  | < 0.001 |
| Married | 1473 (50.3) | 301 (42.9) | 353 (46.7) | 392 (54.2) | 427 (57) |  |
| Widowed | 74 ( 2.5) | 17 (2.4) | 16 (2.1) | 16 (2.2) | 25 (3.3) |  |
| Divorced | 234 ( 8.0) | 46 (6.6) | 59 (7.8) | 63 (8.7) | 66 (8.8) |  |
| Separated | 106 ( 3.6) | 27 (3.8) | 27 (3.6) | 24 (3.3) | 28 (3.7) |  |
| Never married | 705 (24.1) | 229 (32.6) | 203 (26.9) | 153 (21.2) | 120 (16) |  |
| Living with partner | 338 (11.5) | 82 (11.7) | 98 (13) | 75 (10.4) | 83 (11.1) |  |
| PIR | 2.5 ± 1.7 | 2.5 ± 1.7 | 2.5 ± 1.7 | 2.6 ± 1.7 | 2.5 ± 1.7 | 0.594 |
| BMI, (kg/m²) | 28.9 ± 6.9 | 27.1 ± 7.2 | 28.2 ± 7.1 | 29.7 ± 6.7 | 30.3 ± 6.4 | < 0.001 |
| Drinking status, n (%) |  |  |  |  |  | 0.084 |
| ≥ 12 alcohol drinks a year | 1634 (55.8) | 370 (52.7) | 411 (54.4) | 412 (57) | 441 (58.9) |  |
| Smoking status, n (%) |  |  |  |  |  | < 0.001 |
| Smoked at least 100 cigarettes | 1243 (42.4) | 250 (35.6) | 321 (42.5) | 307 (42.5) | 365 (48.7) |  |
| Physical activity (min/week) | 420 (90, 1320) | 450 (120, 1391) | 480.0 (120, 1372) | 360 (60, 1150) | 420.0 (60, 1410) | 0.002 |
| Co-Morbidities |  |  |  |  |  |  |
| Liver condition, n (%) | 102 ( 3.5) | 19 (2.7) | 29 (3.8) | 27 (3.7) | 27 (3.6) | 0.635 |
| Cancer or Malignancy, n (%) | 97 ( 3.3) | 20 (2.8) | 25 (3.3) | 18 (2.5) | 34 (4.5) | 0.137 |
| Serum urea nitrogen, (mg/dL) | 12.2 ± 4.4 | 11.6 ± 4.3 | 12.1 ± 4.7 | 12.5 ± 3.9 | 12.8 ± 4.6 | < 0.001 |
| Serum phosphorus, (mg/dL) | 3.7 ± 0.5 | 3.8 ± 0.6 | 3.7 ± 0.6 | 3.7 ± 0.5 | 3.7 ± 0.5 | 0.011 |
| Serum total protein, (g/dL) | 7.2 ± 0.4 | 7.1 ± 0.4 | 7.2 ± 0.5 | 7.2 ± 0.4 | 7.2 ± 0.4 | < 0.001 |
| Serum uric acid, (mg/dL) | 5.4 ± 1.4 | 5.0 ± 1.2 | 5.3 ± 1.3 | 5.5 ± 1.3 | 5.9 ± 1.4 | < 0.001 |
| Calcium (mg) | 960.3 ± 610.2 | 957.9 ± 588.0 | 956.0 ± 611.2 | 973.3 ± 616.8 | 954.4 ± 624.1 | 0.931 |
| Magnesium (mg) | 306.5 ± 154.0 | 302.2 ± 155.0 | 307.1 ± 160.5 | 304.4 ± 147.1 | 311.7 ± 152.9 | 0.674 |
| Sodium (mg) | 3763.7 ± 1925.6 | 3731.8 ± 1872.3 | 3859.3 ± 1972.9 | 3687.5 ± 1904.7 | 3770.7 ± 1946.4 | 0.362 |
| Potassium (mg) | 2645.2 ± 1252.2 | 2567.9 ± 1235.1 | 2678.0 ± 1300.6 | 2620.9 ± 1213.1 | 2708.1 ± 1253.3 | 0.147 |
| TB-BMD, (mg/cm^2^) | 1111.7 ± 108.0 | 1117.9 ± 107.6 | 1116.2 ± 110.7 | 1108.4 ± 106.6 | 1104.4 ± 106.4 | 0.051 |
| LS-BMD, (mg/cm^2^) | 1031.0 ± 149.6 | 1058.4 ± 149.4 | 1041.3 ± 151.2 | 1019.5 ± 144.0 | 1006.0 ± 148.7 | < 0.001 |
| Serum apolipoprotein B, (mg/dL) | 91.0 ± 25.7 | 60.4 ± 8.9 | 80.2 ± 4.8 | 96.5 ± 5.1 | 125.0 ± 16.9 | < 0.001 |

Categorical variables were expressed as frequencies (%). Variables conforming to a normal distribution were reported as mean (standard deviation, [SD]). Skewed variables were presented as median (interquartile range, [IQR]). PIR, ratio of family income to poverty; BMI, body mass index; TB-BMD, total body bone mineral density; LS-BMD, lumbar spine bone mineral density.
