# Supplementary material for: Association Between Serum Apolipoprotein B and Bone Mineral Density and the Effects of Cardiovascular Disease Mediation: Results From the NHANES 2011–2016 and a Mendelian Randomization Study
Source: Rev Cardiovasc Med. 2025 May 23;26(5):31395. doi: 10.31083/RCM31395 (PMC12135671; doi:10.31083/RCM31395)
Supplement: Supplementary file 1 [file 2153-8174-26-5-31395-s1.zip › Supplementary Table 4.doc]

**Table S4.** Results of univariate analysis of TB-BMD and LS-BMD (mg/cm^2^).

|  | **TB-BMD** | |  | **LS-BMD** | |
| --- | --- | --- | --- | --- | --- |
| **Variable** | **β (95%CI)** | **P-value** |  | **β (95%CI)** | **P-value** |
| Gender, n (%) |  |  |  |  |  |
| Male | Ref |  |  | Ref |  |
| Female | -63.13 (-70.62,-55.65) | < 0.001 |  | 6.12 (-4.74,16.97) | 0.269 |
| Age, (years) | -0.85 (-1.19,-0.51) | < 0.001 |  | -0.93 (-1.4,-0.46) | < 0.001 |
| Race, n (%) |  |  |  |  |  |
| Mexican American | Ref |  |  | Ref |  |
| Other Hispanic | -5.95 (-21.11,9.22) | 0.442 |  | 7.58 (-13.6,28.76) | 0.483 |
| Non-Hispanic White | 19.76 (8.2,31.31) | < 0.001 |  | 36.32 (20.18,52.46) | < 0.001 |
| Non-Hispanic Black | 77.51 (64.49,90.53) | < 0.001 |  | 111.13 (92.95,129.31) | < 0.001 |
| Other Race | -8.22 (-21.81,5.37) | 0.236 |  | 17.17 (-1.81,36.15) | 0.076 |
| Education level, n (%) |  |  |  |  |  |
| Did not graduate from high school | Ref |  |  | Ref |  |
| Graduated from high school | 11.05 (-1.27,23.38) | 0.079 |  | 16.88 (-0.15,33.9) | 0.052 |
| College education or above | 7.12 (-3.3,17.53) | 0.181 |  | 31.21 (16.83,45.59) | < 0.001 |
| Martial status, n (%) |  |  |  |  |  |
| Married | Ref |  |  | Ref |  |
| Widowed | -7.92 (-33.12,17.29) | 0.538 |  | -9.76 (-44.61,25.09) | 0.583 |
| Divorced | -1.55 (-16.43,13.34) | 0.839 |  | 1.94 (-18.65,22.52) | 0.854 |
| Separated | -13.08 (-34.36,8.19) | 0.228 |  | -12.62 (-42.04,16.8) | 0.4 |
| Never married | 11.01 (1.32,20.7) | 0.026 |  | 28.93 (15.53,42.32) | < 0.001 |
| Living with partner | 5.46 (-7.3,18.21) | 0.402 |  | -0.31 (-17.96,17.33) | 0.972 |
| PIR | 2.5 (0.15,4.84) | 0.037 |  | 5.84 (2.59,9.08) | < 0.001 |
| BMI, (kg/m²) | 2.54 (1.98,3.1) | < 0.001 |  | 1.58 (0.8,2.36) | < 0.001 |
| Drinking status, n (%) |  |  |  |  |  |
| ≥ 12 alcohol drinks a year | -10.33 (-18.2,-2.47) | 0.01 |  | -1.65 (-12.56,9.27) | 0.768 |
| Smoking status, n (%) |  |  |  |  |  |
| Smoked at least 100 cigarettes | -0.42 (-8.33,7.5) | 0.917 |  | 14.55 (3.59,25.5) | 0.009 |
| Physical activity (min/week) | 0.01 (0.01,0.01) | < 0.001 |  | 0 (0,0.01) | 0.087 |
| Co-Morbidities |  |  |  |  |  |
| Liver condition, n (%) | 22.22 (0.89,43.54) | 0.041 |  | 47.9 (18.37,77.43) | 0.001 |
| Cancer or Malignancy, n (%) | 22.65 (0.8,44.49) | 0.042 |  | 21.3 (-8.99,51.59) | 0.168 |
| Serum urea nitrogen, (mg/dL) | 1.64 (0.75,2.52) | < 0.001 |  | 0.45 (-0.77,1.68) | 0.47 |
| Serum phosphorus, (mg/dL) | -8.78 (-15.89,-1.67) | 0.015 |  | -2.8 (-12.66,7.06) | 0.578 |
| Serum total protein, (g/dL) | -1.28 (-10.01,7.46) | 0.774 |  | -9.27 (-21.38,2.83) | 0.133 |
| Serum uric acid, (mg/dL) | 15.51 (12.71,18.3) | < 0.001 |  | -0.19 (-4.14,3.77) | 0.927 |
| Calcium (mg) | 0.02 (0.02,0.03) | < 0.001 |  | 0.01 (0,0.02) | 0.003 |
| Magnesium (mg) | 0.09 (0.07,0.12) | < 0.001 |  | 0.05 (0.02,0.09) | 0.003 |
| Sodium (mg) | 0.01 (0.01,0.01) | < 0.001 |  | 0 (0,0.01) | 0.012 |
| Potassium (mg) | 0.01 (0.01,0.01) | < 0.001 |  | 0.01 (0,0.01) | 0.021 |
| Serum apolipoprotein B, (mg/dL) | -0.21 (-0.37,-0.06) | 0.006 |  | -0.73 (-0.94,-0.52) | < 0.001 |

Ref, reference; PIR, ratio of family income to poverty; BMI, body mass index; TB-BMD, total body bone mineral density; LS-BMD, lumbar spine bone mineral density.
