# Supplementary material for: Association Between Serum Apolipoprotein B and Bone Mineral Density and the Effects of Cardiovascular Disease Mediation: Results From the NHANES 2011–2016 and a Mendelian Randomization Study
Source: Rev Cardiovasc Med. 2025 May 23;26(5):31395. doi: 10.31083/RCM31395 (PMC12135671; doi:10.31083/RCM31395)
Supplement: Supplementary file 1 [file 2153-8174-26-5-31395-s1.zip › Supplementary Table 5.doc]

Table S5. After excluding extreme ApoB values results.

| Variable | Unadjusted | | Model 3 | |
| --- | --- | --- | --- | --- |
|  | β (95%CI) | P-value | β (95%CI) | P-value |
| TB-BMD, (mg/cm2) |  |  |  |  |
| ApoB (mg/dL) | -0.25 (-0.41 to -0.09) | 0.002 | -0.27 (-0.43 to -0.11) | 0.001 |
| LS-BMD, (mg/cm2) |  |  |  |  |
| ApoB (mg/dL) | -0.79 (-0.98 to -0.57) | <0.001 | -0.57 (-0.8 to -0.33) | <0.001 |

ApoB, apolipoprotein B; BMD, bone mineral density; TB-BMD, total body BMD; LS-BMD, lumbar spine BMD; Ref, reference.

Model 3 adjust for Model 1+Model 2+serum urea nitrogen, serum phosphorus, serum total protein, serum uric acid, calcium, magnesium, sodium, and potassium.
