# Supplementary material for: Association Between Serum Apolipoprotein B and Bone Mineral Density and the Effects of Cardiovascular Disease Mediation: Results From the NHANES 2011–2016 and a Mendelian Randomization Study
Source: Rev Cardiovasc Med. 2025 May 23;26(5):31395. doi: 10.31083/RCM31395 (PMC12135671; doi:10.31083/RCM31395)
Supplement: Supplementary file 1 [file 2153-8174-26-5-31395-s1.zip › Supplementary Table 6.doc]

Table S6. The detailed information regarding missing data.

|  | Missing value ratio |
| --- | --- |
| Gender | 0 |
| Age | 0 |
| Race | 0 |
| Education level | 7.6% |
| Martial status | 6.0% |
| PIR | 7.6% |
| BMI | 0.3% |
| Drinking status | 12.2% |
| Smoking status | 2.4% |
| Physical activity | 7.5% |
| Liver condition | 7.6% |
| Cancer or Malignancy | 7.6% |
| Serum urea nitrogen | 0.4% |
| Serum phosphorus | 0.4% |
| Serum total protein | 0.5% |
| Serum uric acid | 0.5% |
| calcium | 4.5% |
| magnesium | 4.5% |
| sodium | 4.5% |
| potassium | 4.5% |
