# Supplementary material for: Association Between Serum Apolipoprotein B and Bone Mineral Density and the Effects of Cardiovascular Disease Mediation: Results From the NHANES 2011–2016 and a Mendelian Randomization Study
Source: Rev Cardiovasc Med. 2025 May 23;26(5):31395. doi: 10.31083/RCM31395 (PMC12135671; doi:10.31083/RCM31395)
Supplement: Supplementary file 1 [file 2153-8174-26-5-31395-s1.zip › Supplementary Table 7.doc]

Table S7. The results remained consistent after multiple imputation.

| Variable | Unadjusted | | Model 3 | |
| --- | --- | --- | --- | --- |
|  | β (95%CI) | P-value | β (95%CI) | P-value |
| TB-BMD, (mg/cm2) |  |  |  |  |
| ApoB (mg/dL) | -0.18 (-0.31 to -0.05) | 0.007 | -0.22 (-0.35 to -0.09) | <0.001 |
| LS-BMD, (mg/cm2) |  |  |  |  |
| ApoB (mg/dL) | -0.70 (-0.88 to -0.52) | <0.001 | -0.50 (-0.69 to -0.31) | <0.001 |

ApoB, apolipoprotein B; BMD, bone mineral density; TB-BMD, total body BMD; LS-BMD, lumbar spine BMD; Ref, reference.

Model 3 adjust for Model 1+Model 2+serum urea nitrogen, serum phosphorus, serum total protein, serum uric acid, calcium, magnesium, sodium, and potassium.
