# Supplementary material for: Association Between Serum Apolipoprotein B and Bone Mineral Density and the Effects of Cardiovascular Disease Mediation: Results From the NHANES 2011–2016 and a Mendelian Randomization Study
Source: Rev Cardiovasc Med. 2025 May 23;26(5):31395. doi: 10.31083/RCM31395 (PMC12135671; doi:10.31083/RCM31395)
Supplement: Supplementary file 1 [file 2153-8174-26-5-31395-s1.zip › Supplementary Table 9.doc]

**Table S9.** Sensitivity analysis of serum ApoB on TB-BMD and LS-BMD.

|  | Heterogeneity test | | | |  | Pleiotropy test | | | |
| --- | --- | --- | --- | --- | --- | --- | --- | --- | --- |
|  | Cochrane's Q test | |  | |  | MR-Egger intercept test | |  | |
| apolipoprotein B on | Q | P |  |  |  | Intercept | P |  |  |
| TB-BMD | 343.311 | <0.001 |  |  |  | -0.001 | 0.216 |  |  |
| LS-BMD | 201.974 | 0.010 |  |  |  | -0.001 | 0.352 |  |  |
